# Comprehensive genetic rescreening improves diagnostic yield in congenital hyperinsulinism

**DOI:** 10.1210/jendso/bvag047

**Published:** 2026-03-03

**Authors:** Jonna M E Männistö, Jayne A L Houghton, Jasmin J Bennett, Päivi Keskinen, Tiinamaija Tuomi, Heli Ruuskanen, Liisa A Viikari, Antti Jokiniitty, Jyrki Lähde, Joose Raivo, Timo Otonkoski, Hanna Huopio, Sarah E Flanagan

**Affiliations:** Department of Clinical and Biomedical Sciences, University of Exeter, Exeter EX2 5DW, UK; Kuopio Pediatric Research Unit (KuPRu), University of Eastern Finland, Kuopio FI-70211, Finland; Department of Clinical and Biomedical Sciences, University of Exeter, Exeter EX2 5DW, UK; Exeter Genomics Laboratory, Royal Devon University Healthcare NHS Foundation Trust, Exeter EX2 5DW, UK; Department of Clinical and Biomedical Sciences, University of Exeter, Exeter EX2 5DW, UK; Center for Child, Adolescent and Maternal Health Research, Faculty of Medicine and Health Technology, Tampere University, Tampere FI-33100, Finland; Department of Pediatrics, Tampere University Hospital, Wellbeing Services of Pirkanmaa, Tampere FI-33520, Finland; Department of Endocrinology, Abdominal Centre, Helsinki University Hospital, Helsinki FI-00029, Finland; Folkhälsan Research Centre, Helsinki FI-00250, Finland; Institute for Molecular Medicine Finland FIMM, University of Helsinki, Helsinki FI-00029, Finland; Department of Internal Medicine, Hospital Nova, Wellbeing Services County of Central Finland, Jyväskylä FI-40620, Finland; Department of Pediatrics, University of Turku and Turku University Hospital, Turku FI-20520, Finland; Department of Internal Medicine, Tampere University Hospital, Tampere FI-33520, Finland; Department of Pediatrics, Tampere University Hospital, Wellbeing Services of Pirkanmaa, Tampere FI-33520, Finland; Genome Center of Eastern Finland, Institute of Clinical Medicine, School of Medicine, Faculty of Health Sciences, University of Eastern Finland, Kuopio FI-70029, Finland; Biocenter Kuopio, University of Eastern Finland, Kuopio FI-70029, Finland; Stem Cells and Metabolism Research Program, Faculty of Medicine, University of Helsinki, Helsinki FI-00029, Finland; Pediatric Research Center, New Children's Hospital, Helsinki University Hospital, Helsinki FI-00029, Finland; Department of Pediatrics, Tampere University Hospital, Wellbeing Services of Pirkanmaa, Tampere FI-33520, Finland; Department of Pediatrics, Kuopio University Hospital, Wellbeing Services County of North Savo, Kuopio FI-70029, Finland; Department of Clinical and Biomedical Sciences, University of Exeter, Exeter EX2 5DW, UK

**Keywords:** congenital hyperinsulinism, hyperinsulinaemic hypoglycemia, targeted next generation sequencing, genetic rescreening, children and adolescence, adults

## Abstract

**Context:**

Recent genetic discoveries in congenital hyperinsulinism (HI) and advances in sequencing technology suggest that the diagnostic yield may be improved by rescreening in people with genetically unsolved HI.

**Objective:**

To evaluate this hypothesis in a nationwide cohort of individuals with a historical diagnosis of HI of unknown genetic cause.

**Methods:**

Twenty-seven probands, representing 77% of the genetically unsolved HI cases in Finland, underwent rescreening which targeted the coding regions of 18 known HI genes, and 5 relevant non-coding regions. The median age of the cohort was 21 years (range, 4-44 years). Participants had previously undergone a median of 3 genetic tests (range, 1-4), all of which yielded negative (*n* = 17) or inconclusive (*n* = 10) results.

**Results:**

Genetic rescreening was informative in 22% (6 of 27) of cases. Definitive genetic diagnoses were established in 4 (15%) participants. These included the detection of non-coding variants in the *ABCC8*, *HK1*, and *SLC16A1* genes, and a *GCK* mosaic variant (8% allele fraction). In 2 (7%) cases, rescreening revised genetic results but did not provide a definitive genetic diagnosis.

**Conclusion:**

In this Finnish cohort, rescreening with a comprehensive gene panel provided new or revised diagnoses in 22% of cases, informing on medical management and recurrence risk. These findings emphasize the importance of regularly updating genetic testing strategies and highlight the clinical value of re-evaluating the need for rescreening in genetically unexplained HI cases even following clinical remission.

Congenital hyperinsulinism (HI) is characterized by inappropriate insulin secretion from the pancreatic beta-cells resulting in hypoketotic hypoglycemia and a high risk of brain damage [1-3]. Persistent HI is often monogenic with pathogenic variants identified in over 30 genes [4, 5].

Genetic testing plays a crucial role in the management of drug-unresponsive HI by distinguishing between focal HI, which can be cured through lesionectomy, and diffuse HI, which may require near-total pancreatectomy [1]. Identifying the genetic form of HI can also inform individualized recommendations to prevent hypoglycemia such as dietary or exercise modifications [1, 6, 7], monitoring for extra-pancreatic features [5], and predicting the likelihood of spontaneous remission [8] or future diabetes [9-11]. Importantly, it also enables accurate assessment of recurrence risk and identification of affected family members [12].

Up to 50% of individuals with persistent HI do not receive a molecular diagnosis following genetic testing [7, 13-17]. This is likely related to several factors including yet undiscovered disease genes, incomplete genetic screening, and difficulties with variant interpretation. Interpretation of variants is especially challenging for the most common HI-genes, *ABCC8* and *KCNJ11*, which harbor a high frequency of extremely rare variants and have variable inheritance patterns [18]. This often leads to novel missense changes in these genes being classified as a variant of uncertain significance (VUS) which have no clinical utility.

In a Finnish study of 95 individuals with persistent HI diagnosed over a 40-year period, 32% remained without a molecular diagnosis following targeted next-generation sequencing (tNGS) of 10 HI genes [16]. For these families, and their clinicians, the absence of a genetic diagnosis presents a significant burden of uncertainty and hinders the implementation of personalized management strategies [1, 19].

The understanding of HI genetics is evolving rapidly, driven by ongoing research and advances in genetic technology. Recent discoveries have identified non-coding variants in the regulatory regions of genes such as *HK1, FOXA2*, and *PMM2,* as well as ultra-low-level mosaic variants in genes like *GLUD1* and *GCK* [20-25]. While genetic rescreening, guided by a growing body of genomic knowledge and updated variant interpretation guidelines, has substantially increased diagnostic yield in other rare disorders, it has not been systematically applied to HI [26].

In this study, we report the outcomes of genetic rescreening using an up-to-date, clinically available HI gene panel in a cohort of Finnish individuals with historically diagnosed persistent HI who had undergone varying amounts of genetic testing. We demonstrate that rescreening increases the diagnostic yield and explore its clinical implications in these individuals.

## Materials and methods

### Participants

We recruited 35 individuals with genetically-unsolved, persistent, non-syndromic HI in Finland diagnosed between 1972 and 2024. These individuals were identified by reviewing patient records from all 19 hospitals in Finland treating HI [16], and through a call to clinicians within the national HI network. The diagnosis of HI was based on biochemical evidence of inappropriate insulin secretion during hypoketotic hypoglycemia [1]. Persistent HI was defined as requiring drug therapy for >6 months or pancreatic surgery. Consent for genetic rescreening was obtained from 27/35 probands (77%). The remaining patients were lost to follow-up (*n* = 7) or declined participation (*n* = 1).

### Clinical characteristics

Clinical data of the 27 probands were collected from medical records (Table 1). At the time of this study, the median age of the probands was 21 years (range, 4-44 years). Of those for whom data was available, 12% (3/25) were born large for gestational age (LGA; birth weight and/or length >+2 SDS based on national growth references) [27]. The median age at diagnosis of HI was 60 days (range, 0-878 days), with 44% (12/27) diagnosed during the neonatal period (≤28 days of life).

**Table 1:** Clinical characteristics of the 27 participants with genetically unsolved persistent congenital hyperinsulinism in this study

**Clinical characteristics**	
Male sex	63% (17/27)
Age at the time of rescreening, years	22.1 (4.0-44.1)
HI diagnosis before 2000	33% (9/27)
Born large for gestational age	12% (3/25)
Birth weight SDS	0.5 (−2.6-4.2)
Age at onset of HI, days	60 (0-878)
Neonatal onset of HI	44% (12/27)
Family history of biochemically confirmed HI	11% (3/27)
**Final treatment**	
Diazoxide as final therapy	52% (14/27)
Somatostatin analog as final therapy	22% (6/27)
Combined diazoxide and somatostatin analog	4% (1/27)
Near-total pancreatectomy	19% (5/27)
Partial pancreatic resection for confirmed focal HI	4% (1/27)
**Glycemic outcome at the time of rescreening**	
Currently on HI medication	26% (7/27)
Current diazoxide dose (mg/kg/day)	2.4 (2.0-7.3) (5/27)
Current octreotide dose (mcg/kg/day)	NA (3.0 and 9.4) (2/27)

Categorical variables are presented as % (*n*). Continuous data are presented as median (range). Final treatment refers to choice of effective treatment after possible failed drug trials. Large for gestational age refers to birth weight >2 standard deviation scores.

Abbreviations: HI, congenital hyperinsulinism; SDS, standard deviation score according to national growth references.

The HI had been managed solely with medical therapy in 78% (21/27) (Table 1). At the time of this study, 33% (7/21) of medically-treated probands remained on HI therapy, with a median age of 15.4 years (range, 4.0-26.5 years), while 14 had achieved clinical remission by the median age of 6.4 years (0.5-13.7 years). The remaining 22% (6/27) underwent surgical treatment with all achieving HI remission by a median age of 45 days (range, 11-456 days). This included 5 individuals who had near-total pancreatectomy for suspected diffuse HI, and one who underwent partial resection for histologically confirmed focal HI.

### Statistical analysis

Statistical analyses were performed using IBM SPSS Statistics software version 29.0 (IBM Corp., Armonk, NY). Continuous variables are presented as median (range) and were analyzed using Mann–Whitney U test. Categorical variables are presented as *n* (%) and were analyzed using Fisher's exact test. *P* < .05 was considered significant.

### Previous genetic testing

All 27 probands had undergone a median of 3 rounds (range, 1-4) of genetic testing for HI. In 24 (89%), this included at least 1 test performed as part of a research study (Tables 2 and 3, Table S1) [16, 28]. All but 1 had their most recent genetic test conducted after the publication of the American College of Medical Genetics (ACMG) variant classification guidelines [29].

**Table 2:** The previous and final genetic test results of the 4 (15%) probands with new confirmed genetic diagnoses for congenital hyperinsulinism

Pt. no.	Previous genetic tests (year) [research or commercial]	No. of tests (latest)	Initial result	Final result after retesting
Case 1	SSCP *ABCC8*, *KCNJ11*, *GCK* ex 10, *GLUD1* ex 11-12^*a*^ (2000) [R] SSCP *HNF4A* (2007) [R] tNGS 10 HI genes^*b*^ (2020) [R]	3 (2020)	*ABCC8* p.(Val187Asp), c.560T>A, het, mat, AR (P) (inconclusive)	*ABCC8* p.(Val187Asp), c.560T>A, het, mat (P) *ABCC8* c.1468-52G>A, p.(?) (P), *de novo,* het, on paternal allele
Case 2	SSCP *ABCC8*, *KCNJ11*, *GCK* ex 10, *GLUD1* ex 11-12^*a*^ (2000) [R] SSCP *HNF4A* (2007) [R] tNGS 10 HI genes^*b*^ (2020) [R]	3 (2020)	Negative	*HK1* chr10:g.69348909C>G (P), het, *de novo*
Case 3	Sanger *ABCC8* p.(Val187Asp) and p.(Glu1507Lys) (2009) [C] Sanger *ABCC8*, *KCNJ11* (2010) [C] tNGS 10 HI genes^*b*^ (2020) [R]	3 (2020)	Negative	*SLC16A1* chr1:g.112956380_112956381ins25 bp (P), het, pat
Case 4	SSCP *ABCC8*, *KCNJ11*, *GCK* ex 10, *GLUD1* ex 11-12^*a*^ (2000) [R] SSCP *HNF4A* (2007) [R] tNGS 10 HI genes^*b*^ (2020) [R] tNGS + CNV analysis 9 HI genes (2023) [C]	4 (2023)	Negative	*GCK* p.(Ala454dup); c.1361_1363dup (P) mosaic (8%), het, *de novo*

Transcripts: *GCK*, NM_000162.5; *ABCC8*, NM_001287174.3. Non-coding variants are given according to genomic location (GRCh38).

Abbreviations: AR, autosomal recessive; C, commercial gene panel; CNV, copy number variant; Het, heterozygous; HI, congenital hyperinsulinism; Mat, maternally inherited; P, pathogenic; Pat, paternally inherited; R, genetic testing within research setting; SSCP, single-strand conformation polymorphism; tNGS, targeted next-generation sequencing; VUS, variant of uncertain significance.

^*a*^Methods in [9].

^*b*^Methods in [16].

**Table 3:** The previous genetic test results with final inconclusive genetic diagnosis of congenital hyperinsulinism (n = 10)

Pt.	Previous genetic tests (year) [research or commercial]	No. of tests (latest)	Final genetic test result	Reason for being inconclusive
**Changed genetic result without confirmation of genetic diagnosis (*n*** **=** **2)**				
5	Sanger *ABCC8* p.(Val187Asp) and p.(Glu1507Lys) (2010) [C] tNGS 10 HI genes*^a^* (2020) [R]	2 (2020)	Previously negative. A new *KCNJ11* c.-54C>T, p.(?) het pat VUS identified.	VUS (see Table S2)
6	Sanger *ABCC8*, *KCNJ11* (2010) [C] tNGS 10 HI genes*^a^* (2020) [R]	2 (2024)	Previously identified *KCNJ11* p.(Thr180Ile), c.539C>T, het mat, downgraded from LP to VUS	VUS (see Table S2)
**Previous inconclusive results remained as inconclusive (*n*** **=** **7)**				
7	SSCP *ABCC8*, *KCNJ11*, *GCK* ex 10, *GLUD1* ex 11-12*^b^* (2000) [R] SSCP *HNF4A* (2007) [R] tNGS 10 HI genes*^a^* (2020) [R]	3 (2020)	*ABCC8* p.(Val187Asp), c.560T>A pat, het (P)	No confirmation of focal HI or another recessive variant on maternal allele.
		
		
8	SSCP *ABCC8*, *KCNJ11*, *GCK* ex 10, *GLUD1* ex 11-12*^b^* (2000) [R] SSCP *HNF4A* (2007) [R] tNGS 10 HI genes*^a^* (2020) [R]	3 (2020)	*ABCC8* (p.Val187Asp), c.560T>A pat, het (P)	No confirmation of focal HI or another recessive variant on maternal allele.
		
		
9	SSCP *ABCC8*, *KCNJ11*, *GCK* ex 10, *GLUD1* ex 11-12*^b^* (2000) [R] SSCP *HNF4A* (2007) [R] tNGS 10 HI genes^*a*^ (2020) [R]	3 (2017)	*ABCC8* p.(Leu1552Val), c.4654C>G pat, het (VUS)	VUS (see Table S2)
			
10	SSCP *ABCC8*, *KCNJ11*, *GCK* ex 10, *GLUD1* ex 11-12*^b^* (2000) [R] Seq *HNF4A* (2007) tNGS 10 HI genes*^a^* (2020)	3 (2020)	*ABCC8* p.(Val187Asp), c.560T>A pat, het (P)	No confirmation of focal HI or another recessive variant on maternal allele.
		
		
11	Sanger *ABCC8* p.(Val187Asp) and p.(Glu1507Lys) tNGS 10 HI genes*^a^* (2020) [R]	2 (2001)	*ABCC8* p.(Val187Asp), c.560T>A pat, het (P)	No confirmation of focal HI or another recessive variant on maternal allele.
		
12	tNGS + CNV *ABCC8*, *KCNJ11*, *GCK* [C] SSCP *HNF4A* (2007) [R] tNGS 10 HI genes*^a^* (2020) [R]	3 (2020)	Maternal deletion on chr 11p in the pancreatic tissue consistent with focal HI. Negative germline testing.	Germline variant contributing to focal HI not identified from blood
	
	
13^*c*^	tNGS + CNV analysis 9 genes (2019) [C]	1 (2019)	*ABCC8* p.(Val187Asp), c.560T>A pat, het (P)	No confirmation of focal HI or another recessive variant on maternal allele.
14^*c*^	tNGS + CNV analysis 9 genes (2021) [C]	1 (2021)	*ABCC8* p.(Val187Asp), c.560T>A pat, het (P)	No confirmation of focal HI or another recessive variant on maternal allele.

Transcripts used: *ABCC8*, NM_001287174.3; *KCNJ11*, NM_000525.4 (GRCh38).

Abbreviations: C, commercial gene panel; CNV, copy number variant; Het, heterozygous; HI, congenital hyperinsulinism; Pat, paternally inherited; R, genetic testing within research setting; SSCP, single-strand conformation polymorphism; tNGS, targeted next-generation sequencing; VUS, variant of uncertain significance.

^*a*^Methods in [16].

^*b*^Methods in [9].

^*c*^Exome-based method used for genetic rescreening in this study.

The more historical analyses included single-strand conformation polymorphism (SSCP) analysis and Sanger sequencing targeting 2 *ABCC8* variants commonly reported in the Finnish population, the founder variant p.(Val187Asp), and p.(Glu1507Lys) (based on transcript NM_001287174.3, previously annotated as p.Glu1506Lys using transcript NM_000352.4) [9, 30]. In more recent years, all probands had undergone tNGS of the coding regions of 9 to 14 HI genes with or without copy number variant (CNV) analysis.

Of the 27 probands, 17 (63%) had no variants detected on previous genetic testing. For this study we refer to this as a “negative” result. The remaining 10 (37%) had “inconclusive” results defined as the identification of a variant that could not fully explain HI (Tables 2 and 3, Table S1) [28].

### Genetic analysis and variant interpretation

Testing was performed at the Exeter Genomics laboratory (UK) using DNA extracted from leukocytes (*n* = 26) or saliva (*n* = 1). In 23 probands, tNGS was undertaken using previously described methods [31]. The custom assay captured the coding exons and intron/exon boundaries (−60 bp upstream to +10 bp downstream) of 18 HI genes: *ABCC8*, *CACNA1D*, *CREBBP*, *EP300*, *FOXA2*, *GCK*, *GLUD1*, *GPC3*, *HADH*, *HNF1A*, *HNF4A*, *INSR*, *KCNJ11*, *KDM6A*, *KMT2D*, *MAFA*, *PMM2,* and *TRMT10A*. Additionally, selected non-coding regions of *ABCC8*, *HADH*, *HK1, PMM2,* and *SLC16A1*, where disease-causing variants have been identified, were included in the analysis [6, 20, 23, 32].

Whole exome sequencing (WES) was performed in 4 individuals using a customized Twist Exome Kit (v2.0; Twist Bioscience, San Fransico, USA). Sequencing was conducted using 150 bp paired end reads on an Illumina NovaSeq X Plus system. Bi-directional reads were assembled and aligned to human reference genome (GRCh38/UCSC hg38). Targeted analysis focused on the coding exons and intron/exon boundaries of the 18 genes and 5 non-coding regions listed above.

Both tNGS and WES enabled for the detection of mosaic variants (≥8% allele fraction) and on- and off-target CNVs through read-depth analysis using in-house software SavvyCNV, as described [33]. Prioritized variants were assessed according to the ACMG guidelines and the Association for Clinical Genomic Science Best Practice Guidelines for Variant Classification in Rare Disease [29, 34].

### Family member testing

When a disease-causing variant or a VUS was identified, parental samples were obtained and tested for the variant using Sanger sequencing (details available on request). When a *de novo* variant was identified, parental relationships were confirmed through genome-wide microsatellite analysis (PowerPlex, Promega, Southampton, UK). Co-segregation analysis by Sanger sequencing was performed on additional relatives clinically diagnosed with HI.

### Ethics

The study was conducted in accordance with the Declaration of Helsinki principles with informed written consent obtained from the probands and parent(s) participating in this study. This study was approved by The Regional Medical Research Ethics Committee of the Wellbeing Services County of North Savo [133/2023 (2192/2022)].

## Results

Genetic rescreening obtained an informative result for 6 of the 27 (22%) probands. A new genetic diagnosis was established in 4 (15%), including 3 with a previous negative and one with an earlier inconclusive result (Table 2). In 2 (7%) probands, rescreening led to a revised genetic result but not a definitive genetic diagnosis (Table 3).

### New genetic diagnoses

Pathogenic or likely pathogenic variants confirming a new genetic diagnosis in 4 probands were identified in *ABCC8*, *HK1*, *SLC16A1*, and *GCK* (Table 2). Three of these were in non-coding regions (*SLC16A1*, *HK1*, *ABCC8*) and the coding *GCK* variant was mosaic. All 4 individuals had undergone 2-4 separate genetic tests over a median duration of 27 years (range, 15-39 years).

**Proband 1** is a 38-year-old female previously known to be heterozygous for a maternally inherited, recessive, pathogenic *ABCC8* variant p.(Val187Asp) [16], which on its own could not cause HI. Rescreening identified a second recessive *ABCC8* variant, c.1468-52G>A, p.(?). This previously reported variant is predicted to be pathogenic as it creates a cryptic acceptor splice site, leading to the inclusion of 50 additional nucleotides into exon 10 and a subsequent premature stop codon 31 base pairs downstream [14, 18]. The variant had arisen *de novo*. Phasing analysis using an informative common variant (rs2073587) located on the same sequence read confirmed that the variant was on the paternal allele. This established compound heterozygosity for the 2 recessive variants, consistent with the severe, drug-unresponsive neonatal-onset HI which had required subtotal pancreatectomy at 12 days of age, followed by insulin-dependent diabetes at 9.8 years.

**Proband 2** is a 26-year-old male in whom rescreening identified a *de novo*, heterozygous dominant non-coding *HK1* variant (GRCh38 chr10:g.69348909C>G). This variant has been reported in 19 individuals with HI (as GRCh37 chr10:g.71108665C>G) [20, 21] (Table 2).

The patient was born appropriate for gestational age (AGA) and presented with diazoxide-responsive HI at 3 months (maximum dose 8.1 mg/kg/day). Pancreatic biopsies were taken because initial imaging results suggested an active focus, histological analysis was however consistent with diffuse disease. Diffuse disease was later supported by ^18^F-DOPA-PET/CT imaging which was performed when it was introduced in Finland (Patient 7 in [35]). Despite several attempts to discontinue treatment he remains on low dose diazoxide ∼2.5 mg/kg/day.

**Proband 3** is a 14-year-old-female in whom rescreening revealed a heterozygous 25 bp insertion in the *SLC16A1* promoter [GRCh38 chr1:g.112956380_112956381ins25 bp; previously reported as c.-387_-386ins25 in reference [6] and c.-391_-390ins25 in reference [16]]. This pathogenic variant has been reported in a large Finnish family with exercise-induced HI (EIHI) [6].

The proband was born AGA, presented with neonatal-onset HI, and remains on diazoxide (∼7 mg/kg/day). The variant was inherited from her father who reported adrenergic and neuroglycopenic symptoms of hypoglycemia since prepubertal age but had not undergone formal clinical assessment. A paternal half-sibling with diazoxide-responsive neonatal-onset HI was heterozygous for the variant.

**Proband 4** is a 27-year-old female in whom rescreening identified a dominant in-frame duplication, p.(Ala454dup) in *GCK* (Table 2). This previously reported activating variant [36] was detected at ∼8% (50/617) allele fraction in leukocyte DNA, consistent with post-zygotic mosaicism. Parental testing confirmed the variant had arisen *de novo*.

The proband had neonatal-onset HI with unresponsiveness to initial octreotide therapy (maximum dose 42 mcg/kg/day). A partial, but sufficient response to high dose diazoxide was observed (22 mg/kg/day by 6 months of age) with gradually decreasing dose with age, and discontinuation of therapy at 13.7 years. At 5 years of age ^18^F-DOPA-PET/CT imaging confirmed non-focal disease (patient 6 in [35]).

At the time of this study the patient reported episodes of nonspecific fasting-related symptoms. Following the genetic diagnosis, she underwent a clinical re-evaluation. A supervised fasting test confirmed hypoketotic hyperinsulinaemic hypoglycemia with blood glucose <3.0 mmol/L at 24 hours and <2.8 mmol/L at 32 hours. Medical advice included regular eating, consideration of glucose infusion during significant illnesses, monitoring glucose during possible pregnancies, and consideration of diazoxide if needed.

### Revised results without a confirmed genetic diagnosis

In 2 (7%) individuals rescreening led to changes in the genetic findings, though not to a definitive genetic diagnosis (Table 3, Table S2) [28]. In 1 case with a previous negative result, a previously reported paternally inherited VUS in the promoter of *KCNJ11* (c.-54C>T) was identified [16, 37]. The proband had neonatal-onset HI that resolved by 14 months of age. Focal disease had not been investigated due to diazoxide-responsiveness.

In the second case a heterozygous maternally inherited *KCNJ11* variant, p.(Thr180Ile), previously considered likely to be pathogenic, was reclassified as a VUS, primarily due to insufficient evidence to support a dominant mode of action (Table 3, Table S2) [28]. The proband had diazoxide-responsive HI that remitted by 6 months [16]. The mother was unaffected and the absence of a second *KCNJ11* variant on the paternal allele failed to confirm a recessive disease mechanism.

### Unchanged negative or inconclusive genetic results

In 21 of the 27 (78%) probands, rescreening did not alter the genetic findings. This group included 13 individuals with negative results (Table 3) and 8 with inconclusive results (Table S1) [28].

Among the 8 probands with unchanged inconclusive findings, 6 had a paternally inherited *ABCC8* p.(Val187Asp) recessive variant (Table 3) predicting focal HI which had not been confirmed in any. In 1 individual, an *ABCC8* p.(Leu1552Val) variant inherited from their unaffected father remained classified as a VUS (Table 3, Table S2) [28, 37]. Despite the variant being present in an affected sibling, there was insufficient evidence to support a dominant disease mechanism for the variant. Focal disease was not investigated, and in the absence of a second *ABCC8* variant on the maternal allele a recessive disease mechanism was also not confirmed. The final patient had histologically confirmed focal disease and a previous genetic result reporting a maternal deletion on chr11p in the pancreatic tissue [35]. Rescreening of the patient's leukocyte DNA did not detect a K-ATP channel variant on the paternal allele which would fully explain the focal HI [38].

## Comparison of clinical characteristics

We compared clinical features between individuals with a new genetic diagnosis (n = 4) and those with a negative result following rescreening (*n* = 13) (Table S3) [28]. The only significant difference was a higher median birth weight in the genetically solved group compared to the unsolved group (0.8 SDS vs −0.3 SDS, *P* = .008, respectively). Despite this difference, it is noteworthy that the 4 individuals with a new genetic diagnosis were born AGA. They also tended to have an earlier median age at HI diagnosis compared to the unsolved group (2 days [range, 1-135] vs 183 days [range 0-878 days], respectively) and a greater likelihood of requiring treatment >4 years of age (100% vs 73%) but these did not reach statistical significance (*P* > .05).

## Discussion

In this nationwide Finnish cohort of 27 individuals with genetically unsolved persistent HI, comprehensive rescreening yielded new or revised genetic diagnoses in 6 probands (22%) and 2 affected relatives. Many patients had undergone multiple rounds of genetic testing, often driven by research participation rather than routine clinical care, with the testing strategies employed over the 25-year period reflecting the evolving understanding of HI genetics and advanced sequencing technologies.

In 3 probands with a new genetic diagnosis, variants were identified in the non-coding genome. This included a *de novo* recessive intronic *ABCC8* c.1468-52G>A variant which was not detected by the previous analysis that was limited to +/−10 bp of intron-exon boundaries. In recent years, pathogenic *ABCC8* intronic variants have been described that are located outside the regions typically covered by standard sequencing [18]. These variants are predicted to disrupt splicing by introducing cryptic splice sites or interfering with branch point recognition. The most extreme example is an intronic splicing variant located more than 1000 bp into intron 8 of *ABCC8* [32]. Given the growing recognition of such variants, laboratories should consider whether their sequencing and analysis pipelines can detect deep intronic splicing variants, particularly when a second variant is anticipated.

In 2 individuals, non-coding pathogenic variants were identified in regulatory elements of *SLC16A1* and *HK1.* In the individual with *HK1*-HI, the genomic region had not previously been targeted because the most recent genetic testing occurred prior to the discovery of *HK1* variants as a cause of HI [20]. For the *SLC16A1* variant, although the promoter region had previously been screened, the analysis pipeline used was unable to call the 25 bp insertion [16].

In the final case, a mosaic *GCK* variant was identified at an allele fraction of 8%. While historical SSCP and tNGS within research setting had suggested a change in *GCK*, the variant was not confirmed on Sanger sequencing. A retrospective review of the second more recent tNGS data also confirmed the variant but it had not been called by the diagnostic pipeline due to the allele fractions falling below predefined thresholds.

Low-level mosaicism in dominant HI genes is a recognized cause of disease [24, 25]. Although the level of the *GCK* variant in the pancreas of our patient is not known, it is possible that it is also at a low level in beta-cells, as studies on mice have shown that activating *Gck* variants in a subset of beta-cells can lower the threshold for glucose-stimulated insulin secretion [39]. Because these mosaic variants can occur at frequencies approaching the background error rate of NGS, confirmatory testing is essential to distinguish true mosaic variants from artifacts. In our case, the reproducibility of the finding across methods, together with the exclusion of sample contamination, strongly supports a true mosaic variant.

In 10 individuals, genetic testing yielded either changed or unchanged inconclusive results. This included 3 individuals with a VUS, one of which was identified in this study. Re-evaluation of all variants using the latest interpretation guidelines resulted in 1 variant being downgraded from pathogenic to VUS [29, 34]. The identification of multiple VUS in *ABCC8* and *KCNJ11* was expected given the polymorphic nature of these genes [18] and ongoing challenges in determining whether novel missense variants act in a dominant or recessive manner.

In 6 individuals the unchanged inconclusive result of a paternally inherited *ABCC8* p.(Val187Asp) was insufficient to explain the HI. Possible explanations include small focal lesions that remained undetected on ^18^F-DOPA-PET CT imaging [40] or an undetected maternal variant causing recessive diffuse disease. Although retesting included CNV analysis and limited intronic sequencing, non-coding variants or mosaic variants in leukocyte-derived DNA may still have been missed. Alternatively, given the relatively high allele frequency of this founder variant (∼1:300 individuals among Finnish Europeans in gnomAD v4, and up to 3.5% carrier frequency in certain areas of Finland) [30, 41], the variant may represent an incidental finding unrelated to HI in these patients.

In another case with unchanged inconclusive results, previously identified maternal loss-of-heterozygosity at chromosome 11p in pancreatic DNA was consistent with the observed focal HI, but a corresponding paternal recessive KATP channel variant was not detected in leukocyte DNA [38]. Such a variant may reside in an unscreened region of the KATP channel genes or reflect a second somatic event within the pancreas [42].

In 13 individuals, no genetic cause was identified, consistent with the diagnostic yield reported in other studies [7, 13-16]. Although a monogenic basis could not be confirmed in these individuals, the overlap in clinical features between those with and without a genetic diagnosis suggests that some may harbor undetected variants.

For individuals with genetically unsolved persistent HI, rescreening should balance cost against the likelihood and clinical value of establishing a diagnosis especially as most patients experience spontaneous remission in childhood [8, 43]. In our cohort, 74% had resolved HI including Case 4 with the mosaic *GCK* variant. However, after the genetic diagnosis follow-up testing in this patient confirmed HI after 24 hours of fasting, information that is critical for managing future episodes of hypoglycemia during illness or pregnancy.

Identifying the genetic cause of HI has important implications for reproductive planning not only for the parents of a newborn with HI but also for the patients in whom the HI may have resolved. In Proband 4, germline mosaicism could confer up to 50% recurrence risk, enabling consideration of preimplantation genetic testing or prenatal diagnosis, which may affect pregnancy management or neonatal care. In contrast, confirmation of recessive *ABCC8*-HI in Proband 1 indicates a very low recurrence risk in children.

For others, genetic testing may offer limited benefit. Individuals who have outgrown HI may be lost to follow-up or decline further testing, as seen in 7 cases in this study. These considerations highlight the importance of personalized decision-making and appropriate genetic counseling when evaluating the need for rescreening.

Overall, the results of this study emphasize the importance of regularly updating gene panels to include all recognized genetic causes of disease. The diagnostic yield from this study is consistent with other rare disease rescreening efforts showing that up-to-date testing strategies can uncover previously undetected genetic variants [26]. Importantly, this study ended diagnostic odysseys for individuals after 14 to 38 years since HI diagnosis. These benefits must though be balanced against the possibilities of other findings such as novel or reclassified VUS which may cause uncertainty for families and clinicians tasked with interpreting and managing these inconclusive results.

### Grants or fellowships supporting the writing of the paper

This research was funded in whole, or in part, by Wellcome [223187/Z/21/Z]. For the purpose of open access, the author has applied a CC BY public copyright license to any Author accepted Manuscript version arising from this submission. This research was supported by the National Institute for Health and Care Research (NIHR) Exeter Biomedical Research Centre (BRC). The views expressed are those of the author(s) and not necessarily those of the NIHR or the Department of Health and Social Care. JMEM is the recipient of Fellowships by the European Society for Paediatric Endocrinology (ESPE) and the Finnish Foundation for Paediatric Research (Lastentautien tutkimussäätiö), and the State Research Funding for university-level health research, Kuopio University Hospital, Wellbeing services county of North Savo.

## Disclosures

The authors have nothing to disclose.

## Data Availability

Restrictions apply to the availability of some or all data generated or analyzed during this study to preserve patient confidentiality or because they were used under license. The corresponding author will on request detail the restrictions and any conditions under which access to some data may be provided.
